# Are We Ready for Fragile X Newborn Screening Testing?—Lessons Learnt from a Feasibility Study

**DOI:** 10.3390/ijns4010009

**Published:** 2018-02-13

**Authors:** Tiffany Wotton, Veronica Wiley, Bruce Bennetts, Louise Christie, Bridget Wilcken, Gemma Jenkins, Carolyn Rogers, Jackie Boyle, Michael Field

**Affiliations:** 1 The NSW Newborn Screening Programme, The Children’s Hospital at Westmead, Westmead, NSW 2145, Australia; 2 Disciplines of Paediatrics & Child Health and Genetic Medicine, The University of Sydney, Sydney, NSW 2006, Australia; 3 Sydney Genome Diagnostics—Department of Molecular Genetics, The Children’s Hospital at Westmead, Westmead, NSW 2145, Australia; 4 Genetics of Learning Disability, Hunter Genetics, Waratah, NSW 2298, Australia

**Keywords:** newborn screening, fragile X syndrome

## Abstract

Fragile X syndrome (FXS) is the most prevalent heritable cause of cognitive impairment but is not yet included in a newborn screening (NBS) program within Australia. This paper aims to assess the feasibility and reliability of population screening for FXS using a pilot study in one hospital. A total of 1971 mothers consented for 2000 newborns to be tested using routine NBS dried blood spot samples. DNA was extracted and a modified PCR assay with a chimeric CGG primer was used to detect fragile X alleles in both males and females in the normal, premutation, and full mutation ranges. A routine PCR-based fragile X assay was run in parallel to validate the chimeric primer assay. Babies with CGG repeat number ≥59 were referred for family studies. One thousand nine hundred and ninety NBS samples had a CGG repeat number less than 55 (1986 < 50); 10 had premutation alleles >54 CGG repeats (1/123 females and 1/507 males). There was complete concordance between the two PCR-based assays. A recent review revealed no clinically identified cases in the cohort up to 5 years later. The cost per test was $AUD19. Fragile X status can be determined on routine NBS samples using the chimeric primer assay. However, whilst this assay may not be considered cost-effective for population screening, it could be considered as a second-tier assay to a developed immunoassay for fragile X mental retardation protein (FMRP).

## 1. Introduction

DNA sequencing technology has the potential to change the range of conditions currently screened for using the newborn screening blood spot. To assess conditions, all criteria [1] used for the inclusion of screening tests needs to be evaluated, including the frequency and natural history of the condition understood, the availability of a reliable test suitable for newborn screening, the benefit being reasonably balanced against financial and other costs, and the existence of an established treatment for the condition as well as a satisfactory system in place to deal with diagnostic testing, counseling, treatment, and follow-up of patients.

Fragile X syndrome (FXS, OMIM 309550) is a single-gene disorder on the X chromosome and is the most common heritable cause of cognitive impairment [2], occurring in one in 5000 worldwide [3]. FXS has a significant impact on public health, with affected individuals having a range of complex health challenges from anxiety, sensory integration issues, autism, ear infections, sleep disturbance, seizures, and gastrointestinal problems [4]. It is relatively common compared to some disorders currently included in newborn screening programs.

Fragile X syndrome is predominately caused by an expansion of a trinucleotide (CGG)n repeat present in the 5′ untranslated region of exon 1 of the *FMR1* gene [5]. Affected FXS individuals have CGG triplet repeat expansions of >200 CGG repeats, resulting in gene silencing through methylation and reduced or absent the fragile X mental retardation protein (FMRP) production [6]. Large CGG expansions are typically resistant to PCR amplification and classically have been identified by Southern blot [7]. The (CGG)n repeat is highly polymorphic in the normal population, with normal alleles being considered to have between 10 and 49 CGG repeats. Repeat sizes between 55 and 200 (defined as premutations) have the potential to expand, typically during female transmission to the next generation [8,9]. Repeats of 56 [9] and 59 [8] are the two smallest repeat sizes reported to have expanded during female meiosis into full mutations in a single generation.

In unaffected individuals, the CGG repeat region is interspersed with AGG sequences. Most alleles contain a total of 1–3 AGG interrupts. The frequency and position of AGG interruptions is associated with DNA stability and the risk of expansion in the next generation [10], with fewer AGG interrupts more likely to be associated with expansion.

DNA-based assays are currently used in newborn screening programs as a second tier test on a subset of samples with a positive initial screening test result in order to reduce the number of false positive reports, for example in screening for cystic fibrosis [11]. However, first tier DNA-based assays are starting to be included in screening panels, for example, high-throughput triplex real-time PCR for the screening of severe combined immunodeficiency (SCID) [12]. Even whole-exome sequencing (WES) is being evaluated for inclusion in the not too distant future with the Babyseq study being undertaken to assess the feasibility of DNA sequencing and its impact on newborns and their families [13].

The size of the triplet repeat expansion in individuals with FXS poses technical challenges for a PCR-based newborn screening assay due to the high GC content of the expanded allele, thus resulting in amplification failure. A PCR assay [14], which was modified, uses a FAM-labeled primer (Fu-c) and an unlabeled primer (Fu-f) and can be used on dried blood spot samples. However, females who have a single sized allele by electrophoresis may either be homozygous for a common sized triplet repeat or be compound heterozygotes, having an additional single allele that fails to amplify because it is a large premutation or full mutation. A published PCR assay, which used additional dried blood spot samples collected onto specialized DNA specific filter paper (Whatman FTA Classic, Little Chalfont, UK) and a chimeric CGG-targeted primer, has been reported to allow the rapid and reliable determination of the allele status of all males and females, regardless of whether they have normal, premutation, or full mutation *FMR1* alleles [15].

The use of a screening test and therefore early diagnosis allows relief from a protracted “diagnostic odyssey” and the option to alter future reproductive planning based on this information. These reproductive benefits may extend beyond parents of affected and premutation individuals to include extended family members [16]. Despite easy access to diagnostic testing, the age of diagnosis of FXS has not altered significantly in the last 15 years. A survey of FXS families in Australia indicated a median age of diagnosis for a child with FXS being 5 years [17]. Similarly, in the USA, half of the families have a second child before the diagnosis is confirmed in the first child [18,19].

Benefits of FXS testing may include early access to targeted therapies [4]. It has been reported that the absence of fragile X mental retardation 1 protein (FMRP) leads to the hyperactivation of ERK and mTOR signaling upstream of mRNA translation, affecting cognitive function [20]. Lovastatin, an inhibitor of Ras-ERK1/2, was found to normalize hippocampus protein synthesis in a mouse model, thus restoring brain connections between neurons and improving behavioral symptoms, including hyperactivity. Furthermore, an open label study revealed functional benefits of lovastatin in children and adults [21]. Hence, Ras-ERK1/2 inhibitors may be good candidate therapies.

In addition, matrix metalloproteinase-9 (MMP-9), which is involved in dendritic spine maturation and synapse formation, and is increased in FXS, can be inhibited by minocycline treatment, a tetracycline analogue. An animal study revealed that minocycline promotes the formation of mature dendritic spines and improves behavioral performance significantly at 3 weeks of age in fragile X mouse models [22]. An open label study in humans revealed positive benefits for behavior in adolescents and young adults [23]. Furthermore, a randomized double-blind, placebo-controlled trial of minocycline in children with FXS resulted in greater global improvement than the placebo [24]. Thus, minocycline is currently being used in America to help with behavior problems, anxiety, and attention deficits in children with FXS [4]. Other treatments include metformin, which in a mouse model rescued core phenotypes in *FMR1* knockout mice and normalized ERK signaling, eIF4E (Eukaryotic translation initiation factor 4E) phosphorylation, and the expression of MMP-9, resulting in improvements in behavior and language [25].

Advances in DNA therapy, such as CRISPR-Cas9, have been used to edit CGG repeat expansions in induced pluripotent stem cells and hence, in the future, stem cell therapies may be available for FXS-affected individuals [26].

The aim of this study was to assess the logistic feasibility of adding fragile X screening as an automated first tier assay into a laboratory testing around 100,000 babies per annum and to investigate the impact for follow-up. A prospective pilot study was undertaken to investigate the reliability of a DNA-based first tier newborn screening assay for FXS using a modified chimeric primer method on 2000 samples collected as part of routine newborn screening. A PCR-based fragile X assay designed by Reference [14] was run in parallel with this assay to evaluate the robustness of this approach for newborn screening. Participation rates and attitudes to newborn screening for an untreatable disorder were measured as part of the study and have been reported [27]. This study provides information about allele size in the Australian population, and investigates the stability of premutation alleles detected in this screening cohort using family studies.

## 2. Materials and Methods

### 2.1. Sample Cohort for Study

A total of 2094 women, whose babies were born at the John Hunter Hospital in New South Wales Australia between November 2009 and December 2010, were approached postpartum by a genetic counselor who explained the study. The counselor explained that no additional sample was required as the dried blood spot (DBS) sample already collected for routine newborn screening assays could be used for this test.

**Figure 1:**
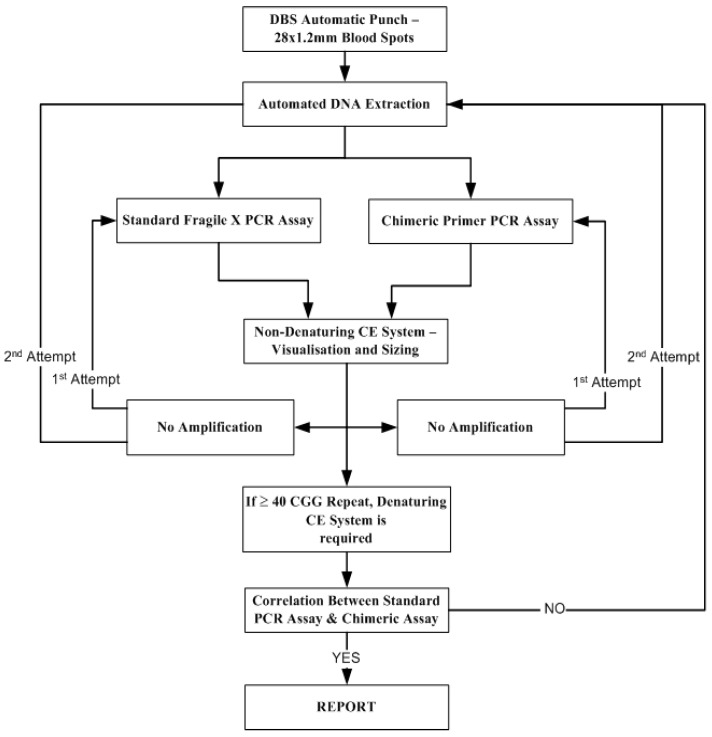
The fragile X pilot testing protocol.

Samples were collected onto pre-printed filter paper cards (Whatman 903, Dassel, Germany) when the baby was 48–72 h of age, allowed to dry, and then forwarded to the NSW Newborn Screening Program for testing, as in Figure 1. Eight normal, four intermediate, four premutation, eight full mutation, and two no DNA DBS control samples were run in every fragile X assay sample plate for quality control.

Please note that if a sample failed to amplify it was re-analyzed using the two PCR assays with the original extracted DNA, and if it failed to amplify a second time DNA was extracted again and then analyzed. In addition, all female samples which produced a single band using the standard PCR assay underwent further testing using Southern Blot analysis to determine whether a given sample was homozygous or compound heterozygote.

### 2.2. DNA Extraction

Genomic DNA extraction from DBS was based on the Qiagen DNeasy Blood and Tissue kit protocol (QA22.doc Oct-01) and modified for use on an automated liquid handler (Epmotion 5075, Eppendorf, Hamburg, Germany). These modifications included setting up a program which incorporated a vacuum manifold instead of centrifuging for the extracting and washing DNA steps. In addition, the liquid handler heating plate was programmed to heat the samples when required. An automated blood spot distributor (BSD 300, BSD, Brendale, Australia) was used to punch various amounts of dried filter paper (1 × 3.2 mm, 3 × 3.2 mm, 1 × 1.2 mm, 28 × 1.2 mm DBS) into a well of a 96-deep-well plate with the optimal results obtained using the increased surface area to volume ratio from 28 × 1.2 mm dried blood spots (equivalent to 12.2 µL of blood) punched as one action, i.e., in one cycle, whereas all other sampling options had variable failure to amplify samples. To avoid potential cross-contamination, each punch head punched 20 blank spots between samples.

Using an automated liquid handler with vacuum and heating block, 180 µL of ATL tissue lysis buffer (Qiagen, Hilden, Germany) was added to each well; the plate was incubated at 90 °C for 15 min, then allowed to cool to room temperature before 20 µL of Proteinase K solution (Qiagen, Hilden, Germany) was added and incubated at 56 °C for 1 h. Then, 200 µL of AL lysis buffer (Qiagen, Hilden, Germany) was added and incubated at 56 °C for 15 min. Next, 200 µL of 100% ethanol (Ajax Finechem, Canning Vale, Australia) was added, and the sample transferred to a filter plate (Qiagen, Hilden, Germany) where the supernatant was extracted by vacuum. The filter bound DNA was then washed with 500 µL of wash buffers AW1 and AW2 (Qiagen, Hilden, Germany), and the DNA was eluted with 150 µL AE elution buffer (Qiagen, Hilden, Germany) and stored at 4 °C. The DNA was tested using both the Fu fragile X assay and chimeric assay. Prior to use in the chimeric primer assay, the remainder of each DNA sample was concentrated to approximately 20 µL in a heating block at 90 °C.

### 2.3. Fragile X Fu PCR Assay

DNA was amplified using a PCR method adapted from Reference [14]. PCR reactions were undertaken using FAM-labeled Fu-c (5′ FAMGCTCAGCTCCGTTTCGGTTTCACTTCCGGT 3′) (Invitrogen, Carlsbad, CA, USA) and Fu-f (5′ AGCCCCGCACTTCCACCACCAGCTCCTCCA 3′) (Invitrogen, Carlsbad, CA, USA) primers and the Expand Long Range PCR system (Roche, Basel, Switzerland). The PCR cocktail consisted of 2 µL of 5× buffer with magnesium, 0.5 µL of 10 mM dNTPs, Deoxyribonucleotide triphosphate, (Astral Scientific, Sydney, Australia), 1.32 µL of 5 µM (5 µM of Fu-c and 5 µM of Fu-f primers) primer mix, 4.4 µL of betaine (Sigma, St. Louis, MO, USA), 0.15 µL of Expand Long Range enzyme mix, and 0.13 µL of water. Using a multi-channel pipette, 8 µL of the reaction cocktail was then transferred to each well and 2 µL of DNA was added. The reaction was covered with 15 µL mineral oil (Sigma, St. Louis, MO, USA) and an adhesive aluminum PCR plate seal. Samples in PCR plates were amplified in a thermal cycler using the pre-programmed cycle listed in Table 1.

All PCR samples were run on the non-denaturing capillary electrophoresis (CE) system (QIAxcel, Qiagen, Hilden, Germany) using the DNA screening gel cartridge kit. Size calculations of the PCR product bands were calculated against nine calibrators, five females and four males, covering a 17–91 CGG repeat range using the QIAxcel BioCalculator Software (version 1.2).

### 2.4. PCR Amplification for the Fragile X Method Using a Chimeric Primer

The method of Reference [15] was modified for reliable amplification of blood collected onto filter paper for routine newborn screening biochemical assays. DNA was amplified using a chimeric CGG-targeted primer coupled with a betaine-based PCR method. The PCR reactions were performed using the FAM-labeled Fu-c primer and (CCG)_4_ chimeric primer (5′ AGCGTCTACTGTCTCGGCACTTGC(CCG) 3′) (Invitrogen, Carlsbad, CA, USA) and Expand Long Template PCR system (Roche, Basel, Switzerland). The PCR reaction mixture A was prepared using 12 µL of 5 M betaine, 4.5 µL of 10 mM dNTPs (Astral Scientific, Sydney, Australia), 1 µL of 10 mM FAM Fu-c primer, and 1 µL of 10 mM (CCG)_4_ chimeric primer. The PCR reaction mixture B consisted of 6.2 µL of water, 3 µL of Expand Long Template buffer 2, and 1 µL of Expand Long Template enzyme mix. Then, 17.5 µL of the PCR reaction mixture A was pipetted into each well of the PCR plate, and 2.5 µL of each DNA sample was added. Wells were covered with 15 µL mineral oil and an adhesive aluminum PCR plate seal. Mixture B (10 µL) was added after an initial denaturation step of 15 min at 99 °C before continuing the thermal cycling (Table 2).

The PCR cycling program reported in Reference [14] was modified as in Table 2. PCR samples were run on a non-denaturing CE system using the high-resolution gel cartridge with a sample injection voltage of 8 kV for 50 s, and a separation voltage of 5 kV for 620 s.

### 2.5. Sizing FMR1 Alleles

All PCR product bands for each sample were analyzed and interpreted, then determined by a second scientist without prior knowledge of the first scientist’s observations. PCR samples from the non-denaturing CE with a fragment size ≥40 CGG repeats were further analyzed using a denaturing CE system as a confirmatory measure, as well as analyzed using Genescan software (version 3.7.1).

Specimens with ≥59 CGG repeats, were retested from the DBS to confirm the sample identity and assess the measurement of uncertainty. All results were issued to the family by staff and provided interpretation and counseling.

The collection of a sample from parents and grandparents of any baby with a result ≥59 CGG repeats was performed to provide the accuracy of that result and confirm allele stability.

### 2.6. Amplidex Assay for AGG Interrupts

DNA was amplified using the Asuragen AmplideX™ *FMR1* PCR Kit and modifications were made, which included increasing the quantity of *FMR1* CGG primer from 1 µL to 2 µL, increasing the concentration of the DNA sample to 50 ng/µL, and excluding diluent from the PCR mix, resulting in improved resolution. This was used to provide information about the presence or absence of interspersed AGG.

The PCR mix consisted of 11.45 µL GC-Rich Amp buffer, 0.5 µL (5 µM of c and 5 µM of f FAM-primers) primer mix, 2 µL of *FMR1* CGG primer, 0.05 µL of GC-Rich polymerase mix, and 1 µL of each DNA sample (50 ng/µL).

## 3. Results

**Figure 2:**
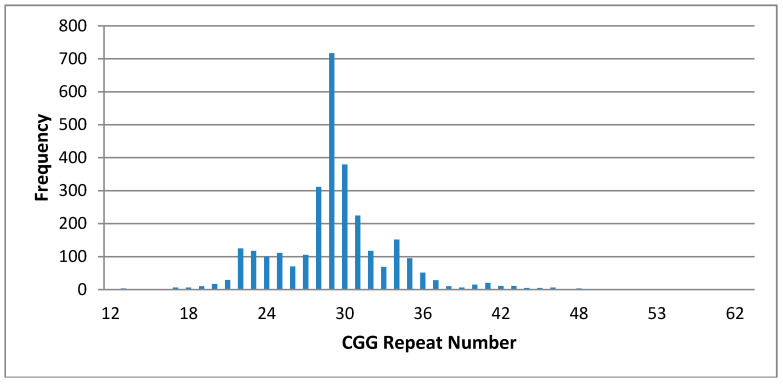
Distribution of CGG repeat sizes obtained from the standard fragile X assay.

Of 2094 women approached by a genetic counselor, 1974 consented for 2003 newborns (1946 single births, 27 sets of twins, and one set of triplets) to be included in the pilot study. Two of these families withdrew consent and three newborns had insufficient blood remaining after routine NBS tests for FXS testing, with one of these family opting not to have a recollection. Therefore, 1971 mothers consented and 2000 newborn screening dried blood spot samples were analyzed, including 987 females and 1013 males. Of the 2000, 1986 had a CGG repeat number below 50 repeats, with the median allele size being 29 (Figure 2).

Ten babies had results of 55–200 repeats, i.e., considered the premutation range, two males and eight females (1/506 males and 1/123 females). No samples had a CGG repeat number in the full mutation range (>200) (Table 3). However, in each sample plate eight full mutation controls were included and identified successfully.

Whilst the pilot study had a 3% retest rate with 56 PCR failures and five extraction failures, all samples provided a result after repeat testing. There was complete concordance between the Fu FXS PCR assay and the chimeric FXS assay for all of the samples analyzed in the pilot study. In addition, a recent review performed by a clinical service for intellectual disabilities, servicing the same region as the birth hospital, showed no clinically identified cases of FXS from this cohort. There were 90 samples with a fragment size ≥40 CGG repeats (Table 3), and these were sent for fragment size analysis.

**Figure 3:**
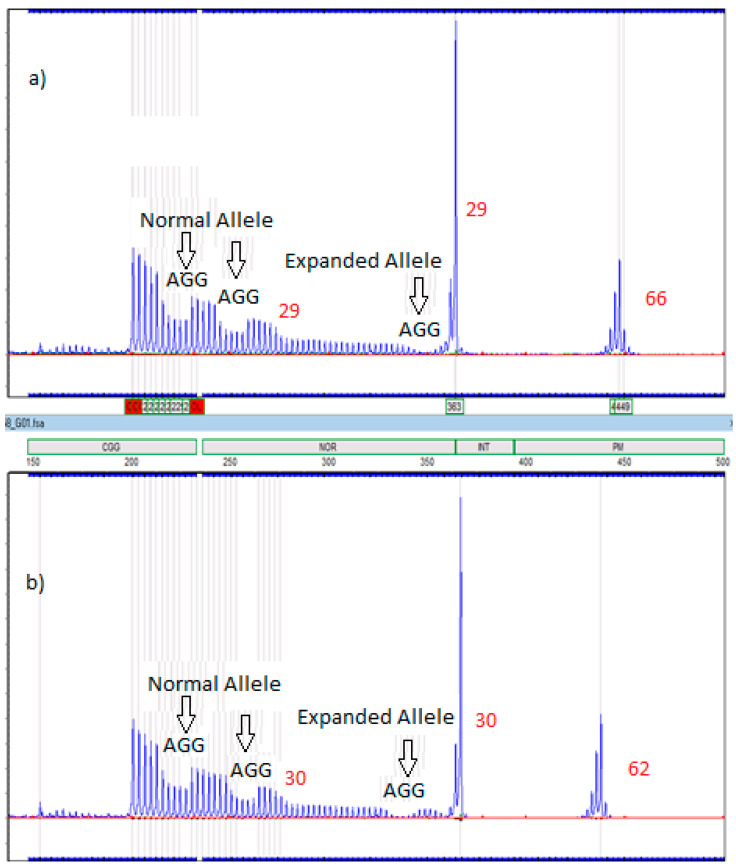
Electropherograms of the proband’s mother (**a**) and the maternal grandmother (**b**) (Case 6) which are 29, 66 CGG and 30, 62 CGG, respectively. They both have two AGG interrupts in the normal allele and one AGG interrupt in the expanded allele.

**Figure A1:**
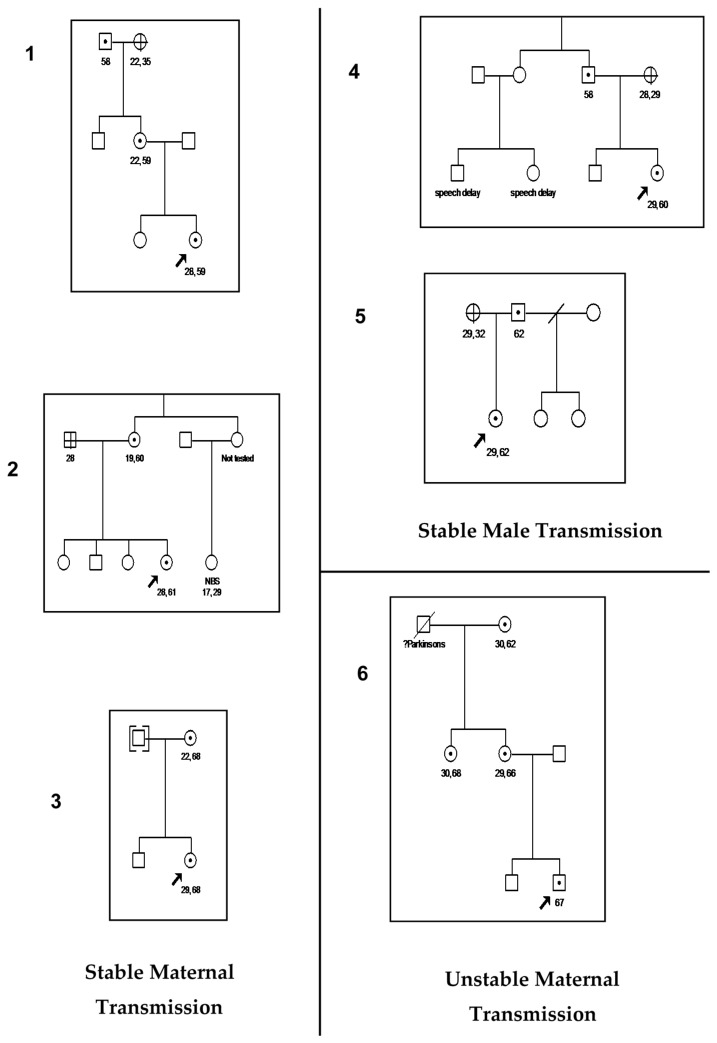
Pedigrees for Cases 1–6.

All samples in the premutation range were at the lower end, from 55 to 68 CGG repeats. The measurement of uncertainty for the lower end premutation reference range was calculated to be ±3 repeats, which was obtained from replicates of samples. For this pilot, six babies were considered to have premutations based on the premutation reference range of 59–200 used by the NSW Molecular Genetics diagnostic laboratory. Two cases were paternally inherited and no change in allele size was demonstrated on transmission, and four cases were maternally inherited. The pedigree with the largest allele (67 repeats), measured across three generations, was the only pedigree to show allele instability, when taking into consideration measurement of uncertainty, with a 62-repeat allele in a maternal grandmother expanding to 66 repeats in the proband’s mother and 68 repeats in an aunt, as well as 67 repeats in the proband (Figure A1—Appendix A). In addition, two AGG interrupts were present in the normal allele and one AGG interrupt was present in the expanded allele of both the maternal grandmother and the proband’s mother (Figure 3) (Table 4).

### Cost of Fragile X Chimeric Assay

The projected cost of screening all babies submitting samples to the NSW NBS Program being tested for FXS using the chimeric assay was also assessed. The cost of performing the fragile X chimeric assay is largely due to the amount of polymerase required for the PCR and the commercial DNA extraction kits. The projected total in-house laboratory expenditure for fragile X screening for NSW would be $1.9 million per 100,000 babies ($19 per baby) each year, or $77,000 per baby ascertained assuming prevalence figures remain constant. The current cost in NSW for all of the routine newborn screening assays, which detects about one in 850 infants with one of the 50 different disorders, is less than AUD$20 per baby or less than $17,100 per case ascertained.

## 4. Discussion

This study confirms the accuracy of the CGG repeat chimeric method when compared to the diagnostic Fu PCR result. Modifications made to the published chimeric method [15] were required to minimize the number of amplification failures from DBS samples and to include a denaturation step before the addition of the enzyme mix and changes in the number of cycles and annealing temperatures. With these modifications and by taking an increased number of 1.2 mm sample aliquots, the analysis could be successfully performed on the routine newborn screening cards. The extra step in the PCR process was not able to be performed using automation, which would be required if the assay were to be used as a population screening test.

Use of the non-denaturing capillary electrophoresis instrument, capable of analyzing 12 samples in one cycle and 96 per run of 96 min, was also modified for the visualization and sizing of PCR fragments for the pilot study. This is an extremely rapid technique when compared with agarose gel preparation previously described [15,28].

In our study we identified one in 124 females and one in 506 males as premutation carriers. Our premutation rates fall close to the premutation rates obtained from an American multi-site screening study, which were one in 209 females and one in 403 males [29]. However, premutation frequencies will differ depending on the specific population. Two Canadian studies revealed premutation allele estimates of one in 800 in males [30] and one in 260 in females [31], whilst in an Israeli study the premutation allele frequency in women was one in 130 [32] and in the Spanish population one in 251 in males [28]. The most common (35%) allele in our population has 29 CGG repeats, with 70% of alleles occurring between 28 to 30 repeats (28 (15%) and 30 (19%) respectively) (Figure 2). This allele distribution is consistent with published studies [28,33]. We did not detect any full mutation individuals in this pilot cohort, which was expected given the reported disease prevalence of one in 4000 in NSW and the fact that disease incidence may have been reduced because of active carrier screening and prenatal testing in known families [34,35].

For the purpose of this pilot study, the patient recall range was defined as greater than 59 repeats. If the range had been defined as 55–200, as used in America [9], there would have been four additional cases requiring further testing (one male and three females). Fragile X carrier testing in the recalled families identified the transmitting parent to be female in four cases, and in three cases there was no evidence of allele instability. In case 6 there was an increase in repeat size from 62 to 66 in one generation and 66 to 67 during the second generation, with the presence of one AGG interrupt in the expanded allele of both the maternal grandmother and the proband’s mother (Table 4). This reduces the risk of expansion to a full mutation in a single generation [36]. Women with 65 CGG repeats and one AGG interrupt had an 11% risk of expansion. However, a small expansion is evident and may eventually become a highly unstable allele.

There is a dilemma to be addressed in the design of any newborn screening program. In the balance of good versus harm, it needs to be assessed whether it is appropriate to detect carriers of a disorder, or only affected individuals. The overwhelming majority of families participating (99%) in this study considered carrier status as useful information and requested this be reported to them [27]; however, concerns about the adult implications of detecting premutation carriers have been raised by some authors [37,38]. Furthermore, the resources required to recall the parents of one in 200 newborns for counseling would be significant, especially if the reproductive impact of this information is limited in many cases. The greatest reproductive benefits occur for higher range premutation allele size and only reporting results of patients with a repeat expansion above 70, where the chance of allele expansion in one generation is 30% or greater, may be an appropriate response. This is further justified by a study [39] that found that women with 60–69 CGG repeats had only a 2% risk of expansion in the absence of a family history. It should be noted that if this were the case, no one would have been recalled in this study.

In addition, supplementary testing of AGG interrupts, as was carried out as part of the family studies or using a PCR assay which primes specifically at the AGG interruptions as described in Reference [40], could be performed on samples which fall within the lower premutation range, 55–70 CGG repeats. This would enable the assessment of allele stability and whether further follow-up is required. Hence, the number of false positives would decrease and this would in turn result in a reduction in clinical costs.

Alternative screening protocols could be considered, with one being based on a methylation specific quantitative high-resolution melt analysis capable of detecting only male and female fragile X-affected patients [41,42]. Another is the incorporation of an immunoassay which quantifies the amount of FMRP present in DBS samples, such as the method described by Reference [43].

The total projected in-house laboratory expenditure for FXS screening for NSW using the chimeric primer assay would be approximately $1.9 million per year, or $77,000 per baby ascertained. When a treatment becomes available, this cost of screening may appear low in comparison to the direct cost to the health system of diagnosis after clinical presentation. However, compared with the current cost of the routine newborn screening assays in NSW of approximately $20 per baby, which includes tests for more than 50 disorders at a cost of less than $17,100 per baby ascertained, it is apparent that this assay in its current format is too expensive for routine newborn screening.

In summary, the fragile X pilot study was conducted to establish the reliability and feasibility of a first tier DNA assay for the newborn screening of FXS. It has demonstrated that the chimeric primer assay can reliably identify, from dried blood spot samples used for routine biochemical screening of other disorders, *FMR1* alleles in the normal, premutation, and full mutation ranges in both males and females. The results of this project provided the first data about the range of intermediate and premutation allele sizes seen in a general community population in Australia. This study also provided insight into the protocol required to provide diagnostic testing, counseling, and follow-up of patients identified through fragile X screening. However, even with automation, the person time and cost required for this assay does not make it feasible for routine newborn screening. Alternative strategies including immunoassays or methylation testing may reduce the cost per sample into a range where a testing program could be considered.

## Figures and Tables

**Table 1:** PCR thermal cycling conditions for standard fragile X assay.

Cycles	Temperature	Time
1 cycle	98 °C	10 min
10 cycles	97 °C	45 s
	64 °C	1 min
	68 °C	4 min
25 cycles	97 °C	35 s
	64 °C	35 s
	68 °C	4 min (+20 s/cycle)
1 cycle	68 °C	10 min

**Table 2:** PCR thermal cycling conditions for the fragile X assay using a chimeric primer.

Cycles	Temperature	Time
1 cycle	99 °C	15 min
Hold (10 μL of PCR reaction mixture B added to each well)	98 °C	
1 cycle	98 °C	2 min
10 cycles	98 °C	35 s
	64 °C	35 s
	68 °C	4 min
27 cycles	98 °C	35 s
	64 °C	35 s
	68 °C	4 min (+10 s/cycle)
1 cycle	68 °C	10 min

**Table 3:** Allele frequencies within the screened population, sized by the standard fragile X assay.

FX Allele CGG Range	Males *n* = 1013	Females *n* = 987
5–49 (*n* = 1986)		
40–44	17	42
45–49	6	11
50–54	1- 52 CGG	3- 31, 51 CGG 29, 52 CGG 30, 53 CGG
55–58 (Premutation: not reported)	1- 55 CGG	3- 29, 55 CGG 31, 56 CGG 29, 57 CGG
59–200 (Premutation: reported)	1- 67 CGG * Unstable	5- 28, 59 CGG * Stable 29, 60 CGG * Stable 28, 61CGG * Stable 29, 62 CCG * Stable 29, 68 CCG * Stable
>200 (Full Mutation)	Nil Found	

* ≥40 CGG repeats resolved by further analysis using a denaturing capillary electrophoresis (CE) system.

**Table 4:** Family studies—AGG interrupt data.

Case Number	Family Member	AGG Number For Expanded Allele
1	Proband’s Mother	2
2	Proband’s Mother	2
3	Proband’s Mother	3
4	Proband’s Father	1
5	Proband’s Father	Insufficient sample
6	Proband’s Mother	1
	Maternal Grandmother	1
